# Application of Third Molar Maturity Index (I3M) for Assessing Adult Age of 18 Years in a Southern Italian Population Sample

**DOI:** 10.1055/s-0042-1744373

**Published:** 2022-06-27

**Authors:** Nino Giannitto, Angela Militi, Daniela Sapienza, Serena Scurria, Patrizia Gualniera, Cristina Mondello, Elvira Ventura Spagnolo, Antonella Terranova, Marco Portelli, Gabriele Cervino, Luca Fiorillo, Aida Meto, Angela Alibrandi, Alessio Asmundo

**Affiliations:** 1 School of Legal Medicine, Department of Biomedical and Dental Sciences and Morphofunctional Imaging, University of Messina, via Consolare Valeria, Messina, Italy; 2 School of Dentistry, Department of Biomedical and Dental Sciences and Morphofunctional Imaging, University of Messina, via Consolare Valeria, Messina, Italy; 3 Multidisciplinary Department of Medical-Surgical and Odontostomatological Specialties, University of Campania “Luigi Vanvitelli,” Naples, Italy; 4 Department of Dentistry, University of Aldent, Tirana, Albania; 5 Endodontic Clinical Section, School of Dentistry, Department of Biomedical and Neuromotor Sciences DIBINEM, University of Bologna, Bologna, Italy; 6 Department of Economics, University of Messina, Piazza Pugliatti, Messina, Italy

**Keywords:** forensic science, forensic anthropology, age estimation, third molar, orthopantomography, immigration, accompanied minors

## Abstract

**Objective** Age estimation of living or dead individuals has a strategic importance in medicine, anthropology, and forensic science, in the context of mass disasters and in civil or criminal matters such as adoption or asylum. Teeth play a major role in this context in particular, the third molars are useful for determining whether an individual has reached the legal age of 18 years because they are still in development from the age of 14.

**Materials and Methods** In this study, a sample of 307 panoramic radiographs performed on healthy subjects aged between 13 and 23 was analyzed to consider the correlation between the maturity index of the third molar (I3M) and age to verify the reliability of the cutoff 0.08 indicated by Cameriere et al in a sample of Italian subjects living in the Province of Messina (Sicily, South Italy) to discern the adult subjects from the minors.

**Statistical Analysis** The analysis of 307 panoramic radiographs resulted in a sensitivity of 89.2% with a confidence interval of 95%, a specificity of 96.5% with a confidence interval of 95%, and a positive predictive value of 96.7%.

**Results** The method proved itself reliable in estimating adulthood in the population of the Messina- Sicily, but the I3M should not be used as the sole indicator to determine whether a person is younger or older than 18 years because age estimation based on dental methods alone has limitations as the third molars suffer from many variations related to their morphology, their location, and their development.

**Conclusion** We recommend a combination of several methods that are available to increase accuracy of age estimation, depending on the different legal requirements in civil or criminal cases.

## Introduction

Age estimation of living or dead individuals has a strategic importance for forensic purposes, and the dental sciences can contribute in this field.

In a mass disaster, for example, an accurate dental age estimation helps to narrow the search for possible victims. Among living people, especially children and adolescents, age estimation is required for civil purposes, such as for adoption processes, or for criminal reasons. In the latter case, it is especially important to estimate the age of majority, and therefore imputability (in chronological order), when a person is suspected of having committed a crime and does not have a personal identity document or claims to be minor.

The problem concerning age estimation in individuals without identity documents who claim to be minors is very current in Sicily and the Mediterranean in general due to migration flows from the North African coasts that often require the help of age estimation methods to determine the right of asylum or expulsion, because in Italy the expulsion of foreign minors is prohibited according to the art.19, co.2, letter a), D.Lgs. 286/98. 1 2

According to the law n. 47/2017 in addition, the principle is affirmed according to which unaccompanied foreign minors are given equal treatment with minors of Italian or European Union citizenship; there is also the absolute prohibition of refoulement at the border and that the expulsion measure can be adopted only on condition that it does not involve “a risk of serious harm for the minor.” Over 200,000 unaccompanied foreign minors, fleeing conflict, persecution or violence, have applied for asylum in Europe in the last 5 years, but it is likely that the number of children who have arrived is much higher; many of them, in fact, are forced to a shadowy existence in Europe, and are at risk of exploitation and abuse. Children and adolescents who travel alone or with their families have specific rights and needs and must first of all be guaranteed safety and protection. On the contrary, despite some important steps forward such as the adoption by Italy of the “Zampa Law” (Law 47/2017) on the protection and reception of unaccompanied minors, the European Union and the member states have responded with increasingly restrictive and dangerous measures, says the organization.

In Italy, it should be noted that the current increase in arrivals by sea in Lampedusa also involves many unaccompanied minors, 2,168 from the beginning of the year to August 31, and families with children, on which the very serious overcrowding of the hotspot and the extending the transfer times to reception centers suitable for hosting them risks having a highly negative impact. Most of the more than 200,000 unaccompanied minors who arrived in Europe to seek asylum come from Afghanistan, Syria, and Eritrea and end up staying in Germany, Greece, Italy, and Sweden. Nonetheless, out of a total of around 35,000 relocated asylum seekers from Greece and Italy over the past 5 years, only 823 were unaccompanied minors. Meanwhile, sea arrivals to Greece almost doubled between 2018 and 2019 (from 32,000 to 60,000 people).

Several methods are widely used for dental age estimation 3 4 and recently a new approach was suggested via evaluation of the third molars (3rdM) because they are the last teeth to develop 5 and they can offer information concerning legal adulthood.

An investigation performed by Cameriere et al 6 verified the plausibility of dental age estimation using solely 3rdM among Caucasians. So far, no data has used this technique in the population of Messina, Sicily. Therefore, the objective of this study was to ascertain the reliability of the method in a sample of healthy Southern Italian subjects living in the Province of Messina (Sicily), using the 3rdM maturity index (I3M) and in particular the cutoff 0.08 indicated by Cameriere et al. In the method based on the 3rdM index, it is instead possible to determine a cutoff, a value beyond which subjects are considered adults; the value is suitably fixed to obtain the lowest possible number of false positives. Based on the observation of the measurements made in this first study, the value chosen as the discriminating limit is 0.08: this means that when the I3M is less than 0.08, it is considered that the subject is at least 18 years old.

The sensitivity of this value is 70% and the specificity of this value is 98%, while the correct classification of subjects is 83%. 6

## Materials and Methods

For this study, 307 orthopantomographs (OPG) of 153 male and 154 female subjects (from the age of 13 to the age of 23), living in the Province of Messina and native of eastern Sicily, performed for diagnostic purposes in the Department of Biomedical Sciences, Dental and Morphological and Functional Images of the University Hospital Gaetano Martino of Messina from 2013 to 2016, were evaluated retrospectively (see Table 1 ). The study was conducted in accordance with the ethical standards laid down by the Declaration of Helsinki (Finland). People with congenital and endocrine diseases were excluded from the study.

**Table 1:** Sample sex frequency

	Frequency	Percentage (%)
F	154	50.2
M	153	49.8
Total	307	100.0

Data confidentiality was preliminarily protected at the time of data collection, in compliance with the European Union Directive 95/46/EC and Italian Legislative Decree 30 June 2003 n. 196; OPGs were performed by a odontologist and catalogued numerically to be examined according to the numeric identification code of the database, showing for each Orthopanoramic X-RAY (OPT) sex, the date of birth, and the date on which the investigation was performed. Finally, the sample was made anonymous through a randomized substitution of the identification code with a progressive number from 1 to 307.

**Fig. 1:**
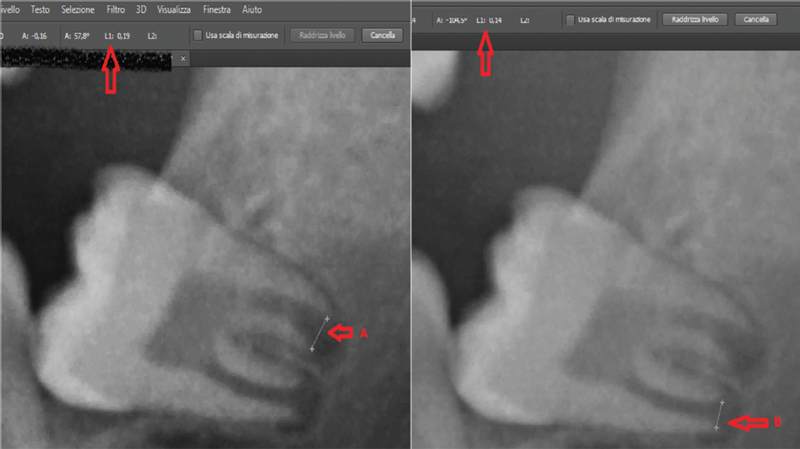
Radiographic measurements.

**Fig. 2:**
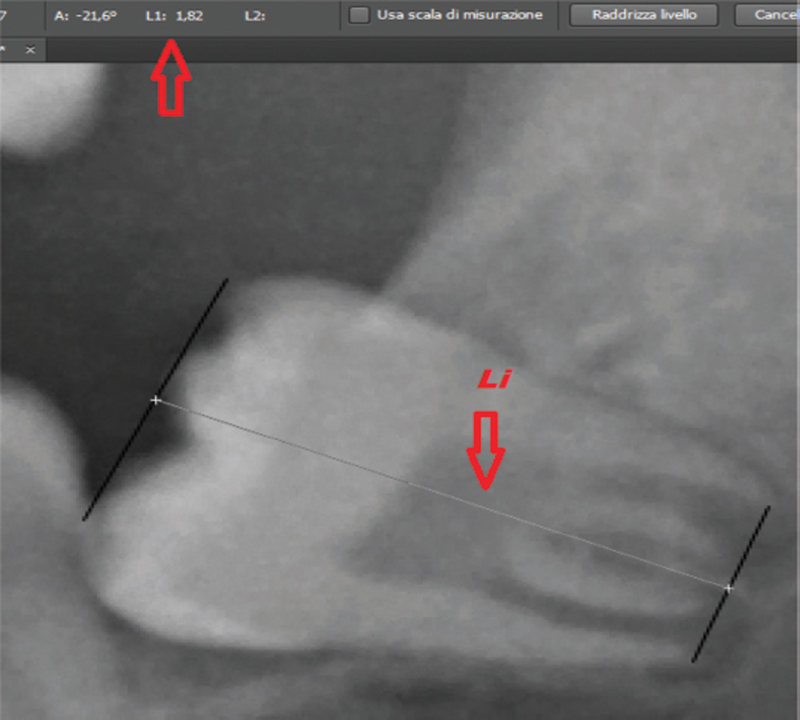
Radiographic measurements detail.

As in the original technique, 6 left lower third molars (LL3rdM), whether impacted or not, were included in the sample if the roots were visible. The criteria for the exclusion from the study were suspicion of congenital and endocrine diseases, dental diseases in the area of interest, agenesis and deformity of the 3rdM, hypodontia. Radiological images, obtained thanks to the orthopantomograph Orthophos Sirona, were saved in JPEG format and were analyzed using the program Adobe Photoshop 2015 using an automatic millimeter-scale, as indicated in the literature by Cameriere et al, 6 measuring the distance between the open apexes of the medial root and of the distal root of the LL3rdM ( Fig. 1 ) and the tooth height, drawing a straight line using the “line” tool that combines the two highest points of the tooth and a line connecting the two lowest points of the tooth and then combining, using the “ruler” tool, the centers of the two lines ( Fig. 2 ). All measurements were performed with the same software.

To determine whether the individual was under or over 18 years old, Cameriere et al's technique 6 verifies the root apex of the LL3rdM of each individual and establishes the I3m: if the LL3rdM presented completed root development (root apex closed), I3M = 0; if the root apex was not completed, the I3M was evaluated as being the sum of the distances of the inner sides of the open apexes (A + B) divided by the tooth length (Li). According to Cameriere et al's cutoff value, an individual is considered to be 18 years of age or older if the I3M < 0.08.

According to the results obtained by these measurements, the I3M as indicated by Cameriere et al was calculated using Microsoft Excel 2007 for Windows and the value obtained was reported later on the file where the OPGs were previously catalogued ( Table 2 ). Statistical analyses were performed using SPSS for Windows, version 17.0. A *p* -value <0.050 was considered statistically significant. The R ^2^ , or coefficient of determination, is a measure of the goodness of fit (or fitting) of the estimated linear regression to the observed data. The R ^2^ does not measure whether there is actually a relationship (of any kind) between the y ^I^ and the regressors, but only to what extent a linear model allows to approximate the reality of the observed data. A nonlinear model, for example, could better represent the relationship between the dependent variable and the regressors, and present a good explanatory power, even in the presence of an R ^2^ close to zero. It is possible to show that adding regressors to the model can only increase the value assumed by R ^2^ ; this does not mean that the model is better. The normality test was applied to only two numeric variables available, I3M and age, using the Kolmogorov–Smirnov test and a *p* -value of 0.001 for the age and of 0,000 for I3M were obtained; this shows a considerable difference between the distribution of the variables examined and the normal one, since the *p* -values are significant (<0.050) ( Table 3 ).

**Table 2:** Sample details. The sensitivity of the test (i.e., the proportion of subjects who are 18 years of age or older who have I3M < 0.08) and its specificity (i.e., the proportion of subjects younger than 18 years of age who have I3M ≥ 0.08) were evaluated, as well as the probability of being 18 years of age or older (when I3M < 0.08)

Name	Date of birth	Date on which the investigation was performed	Age	Sex	I3M
Subject 1	22/03/02	11/06/15	13	F	1.29
Subject 2	06/12/02	23/05/16	13	F	0.97
Subject 3	18/11/92	09/08/06	13	F	0.90
Subject 4	19/09/02	17/11/15	13	F	0.88
Subject 5	09/07/96	13/01/10	13	F	0.86
Subject 6	17/04/93	10/08/06	13	F	0.80
Subject 7	17/04/99	29/01/13	13	F	0.75
Subject 8	17/09/90	20/04/04	13	F	0.72
Subject 9	21/04/98	31/01/12	13	F	0.69
Subject 10	06/12/01	12/06/15	13	F	0.60
Subject 11	11/01/01	04/11/14	13	F	0.58
Subject 12	24/06/00	18/11/13	13	F	0.57
Subject 13	14/02/95	25/03/08	13	F	0.50
Subject 14	04/09/98	10/01/12	13	F	0.40
Subject 15	26/07/00	05/07/14	13	F	0.36
Subject 16	06/08/99	30/10/12	13	F	0.35
Subject 17	21/05/02	27/10/15	13	M	1.3

Abbreviation: I3M, third molar maturity index.

**Table 3:** Kolmogorov–Smirnov test

	Age	I3M
Z of Kolmogorov–Smirnov	1.955	4.603
Asint. Sig. with 2 tails	0.001	0.000

Abbreviation: I3M, third molar maturity index.

## Results

Table 3 shows that, of the 307 OPGs examined, 50.2% concerned female subjects, whereas 49.8% concerned males, while Table 4 shows the number of OPGs in correlation to the sex and age of the subjects.

**Table 4:** Sex frequency and total

Age	Sex		Total
	F	M	
**13.00**	16	15	31
14.00	11	14	25
15.00	15	12	27
16.00	15	13	28
17.00	13	17	30
18.00	6	11	17
19.00	27	12	39
20.00	11	15	26
21.00	14	18	32
22.00	17	12	29
23.00	9	14	23
**Total**	**154**	**153**	**307**

Table 5 shows the results of descriptive statistics concerning numeric variables I3M and age, showing, with regard to age, a mean of 17.98 years (approximated to 18 years), a median of 18 years and a standard deviation of 3.14 and, as regards the I3M, a value between 0.00 and 1.50, a mean of 0.22, a median of 0.08, a standard deviation of 0.29.

**Table 5:** Statistical data

	Mean	Median	SD	Min.	Max.
Age	17.9870	18.0000	3.14671	13.00	23.00
I3M	0.2284	0.800	0.29987	0.00	1.50

Abbreviations: I3M, third molar maturity index; SD, standard deviation.

Table 6 shows the number of female and male subjects belonging to a particular I3M class with the respective mean and standard deviation and *p* -value for each class of I3M obtained by the Mann–Whitney U test that was found to be higher than 0.050, showing no significance between the ages of males and females who are thus essentially overlapping for each I3M class. In Table 6 , you can also note how the age tends to decrease as the I3M value increases.

**Table 6:** Statistical data and standard deviation

I3M	Number of women	Mean of age ± SD	Number of men	Mean of age ± SD	*p* -Value
0.00–0.04	56	20.75 ± 1.63	65	20.86 ± 1.76	0.584
0.04–0.08	24	19.83 ± 1.34	17	19.29 ± 1.04	0.221
0.08–0.3	28	16.46 ± 1.20	33	16.30 ± 1.13	0.556
0.3–0.5	17	14.88 ± 1.21	12	15.00 ± 1.59	0.856
0.5–0.7	10	14.70 ± 2.31	9	14.11 ± 0.92	0.931
0.7–0.9	16	14.06 ± 1.18	12	13.58 ± 0.9	0.280
>0.9	3	13.33 ± 0.57	5	13.20 ± 0.44	0.693

Abbreviations: I3M, third molar maturity index; SD, standard deviation.

Among the participants' images, 148 subjects were found to be true positives (individuals older than or equal to 18 years with I3M <0.08), 5 false positives (individuals below the age of 18 years with 13M <0.08), 18 false negative (subject of age of 18 years or older with I3M ≥ 0.08), 136 true negatives (individuals under the age of 18 years with I3M ≥ 0.08), stratifying individuals by sex ( Table 7 ).

**Table 7:** Statistical data

I3M	Women			Men		
	Age			Age		
	<18	≥18	Tot	<18	≥18	Tot
<0.08	1(b)	74(a)	75	4 (b)	74(a)	78
≥0.08	69(d)	10(c)	79	67(d)	8(c)	75
Total	70	84	154	71	82	153

Abbreviations: a, true positives; b, false positives; c, false negatives; d, true negatives.

According to these data, sensitivity, specificity, positive predictive value, negative predictive value, and the confidence intervals were calculated with diagnostic tests performed on the total sample since a significant difference between sexes has not emerged.

The results showed that, in the examined sample, the test had a sensitivity of 89.2% with a confidence interval of 95% between 0.83 and 0.93, a specificity of 96.5% with confidence interval of 95% between 0.92 and 0.98, a positive predictive value of 96.7% with a confidence interval of 95% between 0.92 and 0.98, and a negative predictive value of 88.3% a confidence interval of 95% between 0.82 and 0.92 and this shows therefore that the probability of identifying a person over the age of 18 years (true positive) is higher than that of identifying a person under the age of 18 years (true negative).

**Fig. 3:**
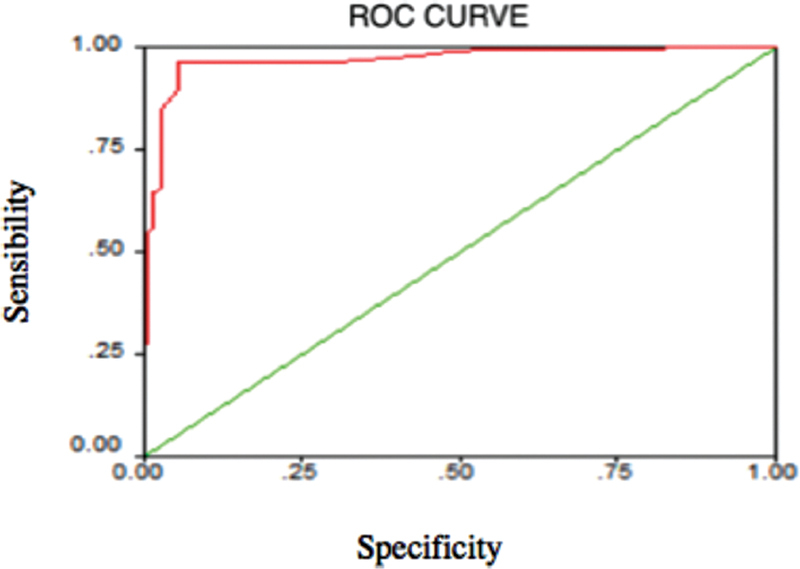
Receiver operating characteristic curve.

Moreover, stratifying diagnostic tests by sex, the test sensitivity in men was 90.2% with a confidence interval of 95% between 0.819 and 0.950, whereas in women it was 88.1% with a confidence interval of 95% between 0.795 and 0.934, whereas the specificity was 98.6% with a confidence interval of 95% between 0.923 and 0.997 in female subjects and 94.4% with a confidence interval of 95% between 0.864 and 0.978 in male subjects. The receiver operating characteristic (ROC) curve ( Fig. 3 ) shows that the “best cutoff” for our sample is the value of 0.08, that is the value that maximizes sensitivity and specificity and that better discriminates between positive and negative subjects. The area under the ROC curve was found to be 0.968, a significance of 0.000 and a confidence interval of 95% for this area between 0.948 and 0.988 ( Table 8 ).

**Table 8:** Statistical data

Area	Standard error (a)	Significance (b)	Asymptotic confidence interval of 95%	
			Lower limit	Upper limit
0.968	0.010	0.000	0.948	0.988

Note: a, according to the nonparametric assumption; b, null hypothesis: real area = 0.5.

A regression was performed ( Table 9 ), considering I3M as the dependent variable and age as the independent variable, and this showed an angular coefficient (B) of −0.074 that is considerable and therefore the I3M depends significantly on age since the *p* -value is less than 0.050, and is also possible to affirm that it depends negatively on age because it decreases as the age increases.

**Table 9:** Regression

Model		Nonstandardized coefficients		Standardized coefficients	*t* -test	Sig.
		Beta	Standard error	Beta		
**1**	(Constant)	1.560	0.063		24.872	0.000
	Age	−0.074	0.003	−0.777	−21.552	0.000

Note: Dependent variable: I3M.

**Fig. 4:**
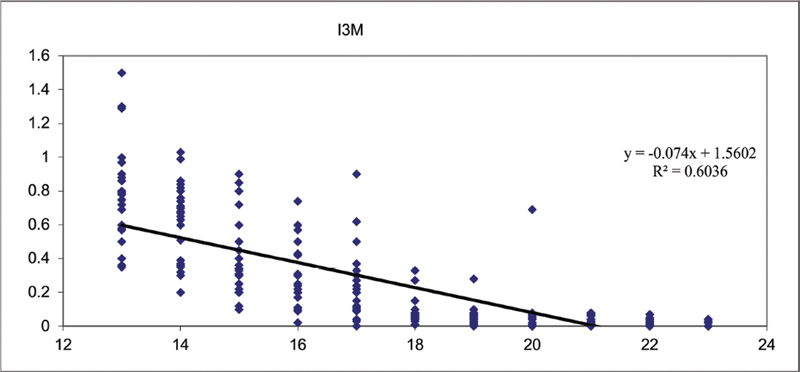
Scatter plot.

According to the results obtained from the regression, a scatter plot was created ( Fig. 4 ). The scatter plot shows the straight line of regression expressed by the equation y = 1.5602 ± + -0.074x, where 1.560 is the intercept of the line and −0.074 is the angular coefficient;

The value of the determination index (R ^2^ ) that is 0.6036 is also reported.

**Fig. 5:**
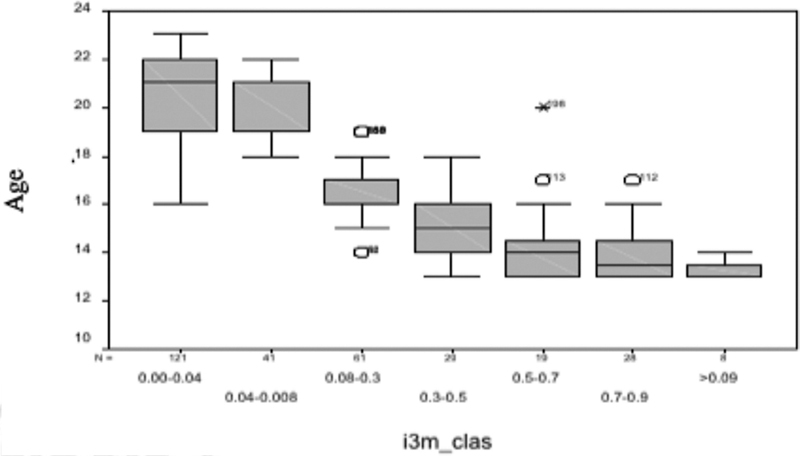
Boxplot.

Fig. 5 shows a boxplot that allows us to observe the trend of the distribution of age for each I3M class in the total sample.

As a chart obtained from a regression, age, as an independent variable, was placed on the horizontal axis, whereas I3M, as the dependent variable, was placed on the ordinate. The segment within some boxes represents the median that is missing in some other boxes because it coincides with the first or the last quartile.

In the I3M class 0.5 to 0.7 values were insufficient to create a box.

The values 186, 56, 198, 113, 112 indicated on the chart represent the so-called “outliers” or values that differ significantly from other observations.

## Discussion

Age estimation (in relation to biological age) via the study of the third molar “maturity” has been very reliable to verify if a person is or is not 18 years old. For identification purposes, the age estimation on corpses and skeletons in forensic medicine has a long tradition, while the application on living subjects constitutes an area of recent investigation, which is becoming increasingly important. In the living, in fact, the estimate of the age can, for example, be relevant to establish the status of a minor individual, in cases of criminal responsibility, asylum requests, child pornography, or adoption. For the first time, the identification of age became a concrete requirement with the industrial revolution; in fact, in that period there was a need to know the age of children to regulate their access to work in factories; however, it is with the impulse of scientific development, on the one hand, and with the protection of human rights on the other, that the approach to research in this specific sector has radically changed. In our society, the age data are fundamental: access to the world of school and work is regulated by age: you must be 18 to be able to vote and to obtain a driving license; these are just a few examples, but there are many disciplines based on the age of the subject. In the legal world, to be recognized as adults, subjects must have completed 18 years of age in Italy; it is the same for many other European countries, but in the world the situation is not homogeneous. The method is noninvasive, easy to perform; in fact, this study is based on a retrospective analysis of OPT performed for other reasons to patients who went to the clinic. From the technique based on the measurement of the open apices of the teeth derives this particular application of the method, which involves taking into account only the 3rdM. 6 7

In fact, in subjects over 12/14 years of age, at the end of the completion of the formation of the second molar, the only dental element still in formation (and therefore assessable for the purposes of estimating the age) remains the 3rdM.

With this technique, we proceed with the calculation of the I3M obtained from the ratio between the opening of the apexes and the height of the tooth.

In practice, when the development of the tooth concerns only the crown, the I3M will be obtained by relating the measure between the two extreme points of the lower margin forming the crown with the maximum height reached by the tooth, measured along the axis of the tooth. While when the development has already reached the roots, the I3M will be obtained from the ratio between the sum of the open apexes of the roots and the height reached by the tooth, measured along its axis. 7

As with other techniques, the measures taken into consideration are related to each other; this allows you to control the effect due to any distortions of the image.

The tooth examined is generally the LL3rdM (3.8) (tooth 38 according to the classification of the FDI -Federation Dentaire Internationale-), but if it is not clearly visible or shows some problems, the contralateral tooth can also be used; the upper molars are never taken into consideration for two main reasons: because they have an even greater variability in development and because they are never clearly visible in OPTs.

In the event that two 3rdM present a notably different development (the right compared with the left), the one with the least development must be taken into consideration (based on the legal principle of presumption of minor age in case of doubt). 8

During childhood, dental age estimation provides more precise results; however, its power decreases as dental development is completed, 7 8 showing a discrete level of confidence until the age of 20 years, as indicated by Aboshi et al. 9

Among young adults, there are two radiological methods available to estimate age: the radiologic study of skeletal bones development and the evaluation of the I3M 8 that is used because at the age of 14 years, when all the other teeth are already mature, it is the only tooth that is still maturing and therefore still available to estimate the age of an individual. 10

Once the wisdom teeth have completed the formation of their roots, the dentition has reached complete and definitive maturation, but some changes continue within the dental elements throughout the course of life. In particular, the internal calcified part of the tooth becomes increasingly larger, making the space available for the soft tissues of the tooth less and less. The “pulp chamber” and the “root canals” become smaller and smaller, which can be easily investigated with a simple endo oral radiograph. We must consider that each person's development can be affected by hormonal, genetic, environmental, nutritional and climate factors or that, according to some authors, tooth development seems to be influenced by exogenous factors such as malnutrition 8 and psychic stress, 8 11 and it is rarely influenced by the presence of pathological conditions. 12

Dental age estimation can also be important in legal issues related to judicial proceedings 13 14 for education, social benefits, employment, and marriage issues. 14

Despite the reliability of the 3rdM as an indicator in age estimation was evaluated by several studies, there is not a consensus on its usefulness 15 and, therefore, on its extensive application in cases of controversial identification of major–minor age of a subject.

It must be said, in fact, that age estimation based on dental methods has limitations, since the 3rdM is subject to many variations linked to its morphology, its position, and its development, 7 and therefore I3M should not be used as the sole indicator to state a majority at 18 years of age. 16

There are also differences between the various populations, between the different methods used for the study of this tooth, and between observers that represent additional barriers. 16 17 18

However, considering all the possible limitations, I3M proved to be able to provide a good accuracy in discerning the individuals under the age of 18 years from the older ones. 11 19 20 21

It should be remembered also that there are recommendations for age estimation as those of AGFAD (Study Group on Forensic Age Diagnostics) or those of the Forensic Anthropology Society of Europe (FASE); they state that age should be estimated by a forensic expert and that the examination should include a physical examination, a radiography of the no dominant side hand, a dental exam (dental condition and ortopantomographs), and a radiography of medial epiphysis of the clavicle. 11 12 22 23 24

There is a discordance in the literature regarding the sex difference, in fact, according to some authors it should be considered, since dental mineralization would be premature for men, 25 whereas according to other authors, there are not any particular differences between the two sexes. 26

In this study, a sample of 307 OPGs performed on healthy Italian subjects, 13 to 23 years old, was analyzed considering the correlation between the I3M and age to verify the applicability of the cutoff 0.08 indicated by Cameriere et al 6 in our sample to discern the adult subjects from minor ones.

Determining whether an individual is older or younger than the age of 18 years, the positive predictive value, sensitivity, and specificity must be associated 10 and Cameriere et al, in the original study, 6 analyzing a sample of 906 Caucasian subjects, obtained a positive predictive value of 83%, with a sensitivity of 70% and a specificity of 98%.

Another study conducted on a sample of Italian subjects, 26 using the same method, 6 has documented a sensitivity of 86.6%, a specificity of 95.7%, and a positive predictive value of 91.4%.

In a study conducted on a sample of Brazilian subjects, 27 the sensitivity was of 77.4%, specificity was of 86.2%, and positive predictive value was of 87.4%, showing values lower than the previous study 26 but higher than the original study. 6

These differences may be due to the fact that in the two previous studies only samples of Caucasians were examined, whereas in Brazil 43.1% of the population is declared as mixed. 28

Our investigation has documented a sensitivity of 89.2% with a confidence interval of 95% between 0.83 and 0.93, a specificity of 96.5% with a confidence interval of 95% between 0.92 and 0.98, a positive predictive value of 96.7% with a confidence interval of 95% between 0.92 and 0.98, and a negative predictive value of 88.3% with a confidence interval of 95% between 0.82 and 0.92 and therefore the probability that a subject living in the Province of Messina with I3M <0.08 is classified as an adult is of 96.5%.

Therefore, a method for age estimation should be accurate, noninvasive and should allow adequate reproducibility 20 and then from an ethical and also legal point of view (Legislative Decree 17.3.1995 n. 230, Implementation of Euratom Directive 80/836, 84/467, 84/466, 89/618, 90/641 and 92/3 on the subject of ionizing radiations), considering that radiological methods, including OPG, expose individuals to “invasive” (potentially health threatening) ionizing radiations, guidelines and international protocols that protect the interests of unaccompanied minors do not allow exposure to ionizing radiations if not for therapeutic purposes, since it does not believe that there is a safe level of radiation, questioning the reliability of these methods. 29 So, the impact that legal measures can have on the life of a person must be considered and, for this reason, it is extremely important to identify the different parameters that can allow to define the major–minor age of an individual. As for forensic purposes, it is necessary to limit as much as possible the number of false positives and false negatives, since a false positive could infringe the protection of children's rights, whereas a false negative could lead to a more lenient “treatment” of a criminal.

However, we have to observe that the application of I3M for assessing adult age of 18 years according to Cameriere et al 6 presents substantial limitations, for example, due to congenital and iatrogenic absence of the 3rdM or due to the inability to identify the ethnicity of the examined subject, especially when the survey is performed in geographical regions where there is a very high level of miscegenation as in Brazil 13 and, for this reason, AGFAD and FASE recommended using this method with caution and in association with other methods for age estimation.

We should bear in mind that the OPG has a bias of 30% and that especially comparing with cone beam computed tomography (CT) in the study of the facial skeleton, 30 it was found that the cone beam provided a more accurate and useful radiographic resolution.

Comparing these results with other studies conducted on Caucasian, 6 Brazilian, 27 Italian, 26 Albanian, 31 and Croatian 32 subjects ( Table 10 ), they are higher in terms of sensitivity and positive predictive value but lower in terms of specificity compared with the study conducted on Caucasian subjects 6 and higher in terms of sensitivity, specificity, and positive predictive value compared with studies on Brazilians, 27 Italians, 26 Albanians, 31 and Croatians. 31 32 33 34 35 36 37

**Table 10:** Statistical data about sensitivity and specificity of the sample

	Sensitivity	Specificity	Positive predictive value
Caucasians 6	70%	98%	83%
Brazilians 27	77.4%	86.2%	87.4%
Italians 26	86.6%	95.7%	91.4%
Albanians 31	84.75%	93.75%	95.8%
Croatians 32	87.9%	93.65%	95.5%

These differences may be due to the fact that in these studies, excluding the one conducted on Italian subjects, 26 only samples of Caucasian, Albanian, Croatian, and Brazilian subjects were examined and in Brazil 43.1% of the population is declared as mixed. 28

Our survey also showed that, although the application of I3M for assessing adult age is reliable for discerning individuals younger than 18 years of age from older ones in our sample, the precision and the accuracy of the I3M decrease when the age increases. Before getting to the heart of the matter, it should be remembered that the maturation times of the skeleton vary according to the sex of the subject: it is known that females presume an earlier development than males, throughout the juvenile period. The specific request for an “X-ray of the left hand,” which has become a customary practice, actually arises in consideration of the fact that in a subject the preferential limb is more subject to stress, to micro traumas, which could alter its development, therefore, considers the district of the non-preferential limb more indicative. This means that before proceeding with the acquisition of the X-ray to be examined, it should be checked whether the subject is right-handed or not and only then can the nonpreferential limb be subjected to radiographic examination.

In practice, however, the analysis of the wrist-hand area of the left side has been implemented as a standard. The demand for ever more precise methodologies for ascertaining the age increases considerably when the subject in the growth phase is toward the terminal phase of skeletal growth. The age group that poses the most problems for researchers is the one associated with age limits that have a legislative value.

In the specific case of European countries, one of these limits is represented by the age of 18.

At this stage, most of the bones have already completed their formation and there are few elements that can allow us to evaluate the skeletal age of the subject, as well as the dental age. The fact that there are few age indicators that can be analyzed to ascertain this particular age strongly clashes with the legal need to define a subject as a minor or as an adult.

Two districts are particularly interesting for estimating the age in these phases: the sternal end of the clavicle and the iliac crest of the ileum. For both, three phases have been identified: the lack of union (the two portions are separated from each other), partial union, and complete union. For the evaluation of the attainment of 18 years or less, it may be useful to evaluate the state of ossification of the medial extremity of the clavicle, given that almost all the skeletal indicators of development have already completed their growth at this stage; this district is in fact considered the last skeletal element to complete its formation.

The radiological methods for examining the medial epiphysis of the clavicle in living subjects are conventional radiography and CT; data on applications with new approaches using magnetic resonance and ultrasound are also available. Magnetic resonance imaging (MRI) has been applied to the analysis of the wrist–hand district in sports, to ascertain the age of players, through the development of a system based on six degrees of fusion.

The applications of MRI to the study of the clavicle should not be forgotten. Ultrasound also constitutes a future perspective in the radiation-free approach that has been evaluated.

The cutoff of 0.08 was confirmed as “best cutoff” by our investigations, but we must bear in mind that I3M cannot be yet used as an exclusive criterion for estimated age especially when the apexes of the roots of the 3rdM are completely closed; if so, it should be associated with other “markers” of age as recommended by AGFAD and FASE. The chronological age does not always coincide with the biological age; a sportsman forty, for example, may have a biological age of 30 (sports have various rules and characteristics that involves the stomatognathic system differently), vice versa, an obese and sedentary 30-old may have a biological age of 40. Similarly, a boy may have early or delayed puberty. Currently, there is no scientific method that allows a certain determination of the chronological age. 38 39 40 41 42 43 44 45 46
